# ‘Utilitarian’ judgments in sacrificial moral dilemmas do not reflect impartial concern for the greater good

**DOI:** 10.1016/j.cognition.2014.10.005

**Published:** 2015-01

**Authors:** Guy Kahane, Jim A.C. Everett, Brian D. Earp, Miguel Farias, Julian Savulescu

**Affiliations:** a Oxford Uehiro Centre for Practical Ethics, University of Oxford, Littlegate House, St. Ebbe’s St., Oxford OX1 1PT, UK; b Oxford Centre for Neuroethics, University of Oxford, Littlegate House, St. Ebbe’s Street, Oxford OX1 1PT, UK; c Department of Experimental Psychology, University of Oxford, South Parks Road, Oxford OX1 3UD, UK; d Centre for Research in Psychology, Behaviour & Achievement, Coventry University, Priory Street, Coventry CV1 5FB, UK

**Keywords:** Moral judgment, Moral dilemmas, Utilitarianism, Psychopathy, Altruism, Impartiality

## Abstract

A growing body of research has focused on so-called ‘utilitarian’ judgments in moral dilemmas in which participants have to choose whether to sacrifice one person in order to save the lives of a greater number. However, the relation between such ‘utilitarian’ judgments and genuine utilitarian impartial concern for the greater good remains unclear. Across four studies, we investigated the relationship between ‘utilitarian’ judgment in such sacrificial dilemmas and a range of traits, attitudes, judgments and behaviors that either reflect or reject an impartial concern for the greater good of all. In Study 1, we found that rates of ‘utilitarian’ judgment were associated with a broadly immoral outlook concerning clear ethical transgressions in a business context, as well as with sub-clinical psychopathy. In Study 2, we found that ‘utilitarian’ judgment was associated with greater endorsement of rational egoism, less donation of money to a charity, and less identification with the whole of humanity, a core feature of classical utilitarianism. In Studies 3 and 4, we found no association between ‘utilitarian’ judgments in sacrificial dilemmas and characteristic utilitarian judgments relating to assistance to distant people in need, self-sacrifice and impartiality, even when the utilitarian justification for these judgments was made explicit and unequivocal. This lack of association remained even when we controlled for the antisocial element in ‘utilitarian’ judgment. Taken together, these results suggest that there is very little relation between sacrificial judgments in the hypothetical dilemmas that dominate current research, and a genuine utilitarian approach to ethics.

## Highlights

• ‘Utilitarian’ judgments in moral dilemmas were associated with egocentric attitudes and less identification with humanity.
• They were also associated with lenient views about clear moral transgressions.
• ‘Utilitarian’ judgments were *not* associated with views expressing impartial altruist concern for others.
• This lack of association remained even when antisocial tendencies were controlled for.
• So-called ‘utilitarian’ judgments do not express impartial concern for the greater good.

## 1 Introduction

According to classical utilitarianism, we should always aim to maximize aggregate welfare (Bentham, 1789, Mill, 1861). Utilitarianism is a radically impartial view: it tells us to consider things as if ‘from the point of view of the universe’ (Sidgwick, 1907), without giving any special priority to ourselves, or to those dear or near to us. Instead, we should transcend our narrow, natural sympathies and aim to promote the greater good of humanity as a whole, or even the good of all sentient beings (Singer, 1979). Needless to say, this view of morality is strongly at odds with traditional ethical views and common intuitions. It is also a highly demanding moral view, requiring us, on some views, to make very great personal sacrifices, such as giving most of our income to help needy strangers in distant countries (Kagan, 1989, Singer, 1972).

A great deal of recent research has focused on hypothetical moral dilemmas in which participants must decide whether to sacrifice the life of one person in order to save the lives of a greater number. In this large and growing literature, when individuals endorse this specific type of harm they are described (following Greene, Sommerville, Nystrom, Darley, & Cohen, 2001) as making *utilitarian* judgments; when they reject it, they are said to be making *non-utilitarian* (or deontological) judgments.^2^ This terminology suggests that such ‘utilitarian’ judgments express the kind of general impartial concern for the greater good that is at the heart of utilitarian ethics. This is a widely held assumption. For example, it has been argued that this research shows that utilitarian judgment is uniquely based in deliberative processing involving a cost-benefit analysis of the act that would lead to the greatest good, while, by contrast, non-utilitarian judgment is driven by instinctual emotional aversion to causing ‘up-close-and-personal’ harm to another person (Greene, 2008). It has even been argued that this empirical evidence about the psychological sources of utilitarian and non-utilitarian judgment can help explain the historical debate between utilitarians and their opponents (Greene, Nystrom, Engell, Darley, & Cohen, 2004) and, more radically, even that it should lead us to adopt a utilitarian approach to ethics (Greene, 2008, Singer, 2005).

However, as we have pointed out in earlier work, these large theoretical claims are problematic. This is because endorsing harm in the unusual context of sacrificial dilemmas need not express anything resembling an impartial concern for the greater good (Kahane, 2014, Kahane and Shackel, 2010). Indeed, the sacrificial dilemmas typically used in current research represent only one, rather special, context in which utilitarian considerations happen to directly conflict with non-utilitarian rules or intuitions. To be willing to sacrifice one person to save a greater number is merely to reject (or overrule) one such non-utilitarian rule. Such rejection, however, is compatible with accepting extreme non-utilitarian rules in many other contexts—rules about lying, retribution, fairness or property, to name just a few examples, not to mention non-impartial moral norms permitting us give priority to ourselves, and to our family or compatriots, over others. Indeed, to reject a specific non-utilitarian moral rule (or even many such rules) is not yet to endorse the core of utilitarianism: the positive aim of impartially maximizing the greater good of all.

It therefore cannot be assumed that a tendency to make ‘utilitarian’ judgments in sacrificial ‘personal’ dilemmas really reflects any kind of genuine concern for the greater good. In fact, two recent studies observed no correlation or even a negative correlation between a tendency to make such ‘utilitarian’ judgments and seemingly genuine utilitarian judgments or attitudes in other contexts. First, in a prior study, we found no correlation between rates of ‘utilitarian’ judgment and utilitarian views in a context in which utilitarian considerations were pitted against rules against lying or disrespecting autonomy (Kahane et al., 2012). Second, clinical populations have been reported to exhibit both higher rates of ‘utilitarian’ judgment in personal moral dilemmas (Koenigs et al., 2007) as well as greater rates of punitive responses to unfair offers in the Ultimatum Game (Koenigs & Tranel, 2007)—retributive responses that are at odds with a strict utilitarian cost-benefit analysis. A ‘utilitarian’ bias in the context of sacrificial dilemmas thus may not carry over to other contexts, casting doubt on the assumption that it is driven by a general concern with maximizing the good.

Even more strikingly, several recent studies found that ‘utilitarian’ judgment is associated with *anti*-social traits such as psychopathy (Bartels and Pizarro, 2011, Glenn et al., 2010, Koenigs et al., 2012, Wiech et al., 2013), as well as with diminished empathic concern (Choe and Min, 2011, Crockett et al., 2010). It seems rather implausible that individuals with antisocial traits or lower levels of empathy are especially morally committed to promoting the greater good, or harbor a special concern for humanity as a whole.

Suggestive as this recent evidence may be, the relationship between ‘utilitarian’ judgment in sacrificial dilemmas and impartial utilitarian concern for the greater good has not yet been examined in a direct and robust fashion. It cannot be ruled out, for example, that some individuals with lower empathy may nevertheless arrive, in a ‘cold’ fashion, at a more general utilitarian outlook. Moreover, even if there is an antisocial component driving some ‘utilitarian’ judgments, it remains possible that, once this component has been controlled for, a pattern strongly associating ‘utilitarian’ judgment and general concern for the greater good will emerge.

The aim of the present study was therefore to directly investigate the relation between ‘utilitarian’ judgment in sacrificial dilemmas and clear markers of impartial concern for the greater good in other moral contexts (e.g. increased altruist concern for distant strangers) and within the context of sacrificial dilemmas (e.g. willingness to sacrifice oneself to save a greater number), as well as their contraries (e.g. support for egoism or greater willingness to sacrifice someone when this also benefits oneself).

Now, if a strong tendency toward ‘utilitarian’ judgment in classical sacrificial dilemmas really reflects giving greater (or even exclusive) priority to impartial promotion of the good of all, or a preference for a utilitarian style of moral reasoning—as implied by much of the current work in this area—then we should expect this tendency to be observable as well in *other* contexts in which impartial utilitarian concern for the greater good competes with self-interest and with other moral concerns.

In contrast, if a tendency to ‘utilitarian’ judgment reflects a narrower moral disposition largely driven, not by concern for the greater good, but by reduced aversion to harming others (Crockett et al., 2010, Cushman et al., 2012), then we would expect *no* association between a ‘utilitarian’ bias in this special context and greater endorsement of paradigmatic utilitarian judgments in other contexts. Moreover, to the extent that such a ‘utilitarian’ bias is in fact driven by antisocial tendencies, we would rather expect a *negative* association between ‘utilitarian’ judgment and markers of genuine concern for the greater good, and a *positive* association with selfish and amoral views and dispositions. Such a pattern of results would cast serious doubt on the common assumption that so-called ‘utilitarian’ judgment in sacrificial dilemmas expresses a general concern for the greater good.

Before we proceed, two clarifications are in order. First, what is at issue here is *not* whether ordinary folk explicitly endorse and consistently follow an abstract utilitarian theory; it is clear that few if any do. What is at issue is whether individuals with a marked tendency to ‘utilitarian’ judgments in sacrificial dilemmas are expressing an outlook that is at least in the broad *direction* of impartial concern for the greater good (Kahane & Shackel, 2010).^3^ It would be too much to expect such individuals to judge, for example, that they must give most of their money to distant strangers as utilitarianism may require. But one would expect them at least to be more inclined than others to judge that we should give *some* of our money to help such people in need. Since such an impartial moral outlook can manifest itself in more than one way, we shall consider a range of possible markers of concern for the greater good.

Second, by impartial concern for the greater good, we mean the utilitarian view that we morally ought to always maximize the aggregate happiness of all. This is primarily a claim about people’s moral *judgments*—their views about what we ought to do. It is not, in the first instance, a claim about motivation or behavior. But although people do not always act on their moral judgments (e.g. they may eat meat despite thinking this is wrong), people’s behavior is often good evidence for their moral judgments. For example, if people give a great deal of their money to charity even if this in no way benefits them, it is highly unlikely that they nevertheless believe that this act is deeply wrong. Thus, although most of the measures we employ relate to moral judgments, we shall also assume that behavior (and predicted behavior) expressing greater-than-average impartial altruism is also strong evidence of greater concern for the greater good. Moreover, although our main focus is on people’s moral views, the relationship between sacrificial dilemmas and utilitarian behavior in real-life contexts is of independent theoretical and practical interest.

## 2 Study 1

Although ‘utilitarian’ judgment in sacrificial dilemmas is widely assumed to reflect a utilitarian concern with the greater good, there is recent evidence, reviewed above, that it is rather driven by reduced aversion to harming (Crockett et al., 2010, Cushman et al., 2012) and associated with antisocial traits (Bartels and Pizarro, 2011, Glenn et al., 2010, Koenigs et al., 2012, Wiech et al., 2013) and reduced empathy (Choe and Min, 2011, Crockett et al., 2010).

One aim of Study 1, therefore, was to replicate this reported association and to disentangle the respective roles of antisocial tendencies and reduced empathic concern in ‘utilitarian’ judgment. More importantly, we wanted to directly investigate the relationship between ‘utilitarian’ judgment and moral judgments in a completely different moral domain, relating to everyday violations of ethical norms in a professional context (e.g. embezzling money)—a domain that does not involve the up-close-and-personal harm central to classical sacrificial dilemmas. Note that whereas classical sacrificial dilemmas aim to contrast two opposing moral outlooks (utilitarian vs. deontological), the business ethics transgressions in question involve self-interested violations of uncontroversial moral norms. In this respect, they assess one’s attitude toward the need to behave morally in general, with low ratings of wrongness expressing a broadly amoral standpoint.

If ‘utilitarian’ judgment really is driven by concern for the greater good, we would expect it to be associated with more severe assessment of the wrongness of such moral transgressions in another context. If ‘utilitarian’ judgment is instead driven by a focused reduced aversion to physically harming others, there should be no correlation between moral judgments across these contexts. However, if ‘utilitarian’ judgment is in fact driven by a broader antisocial tendency, we would expect instead that higher rates of ‘utilitarian’ judgment would be associated with a more lenient assessment of the wrongness of these moral transgressions in a completely different moral context.

### 2.1 Method

#### 2.1.1 Participants and procedure

US participants were recruited via the online service Mechanical Turk (MTurk), and received $0.40 for their time. Participants were excluded from analysis if they did not complete the survey, failed an attention check or if they completed the survey in too short a time to have paid full attention (<300 s). The attention check constituted a question embedded in the survey in which participants were requested to put a certain digit to confirm that they were paying attention. Participants who completed the survey in too short a time to have paid attention were excluded (*N* = 24).^4^ As such, our sample consisted of 194 participants (66 female; *M_age_* = 31, *SD* = 9.49). This study and the following ones were approved by the local Research Ethics Committee.

Participants completed an online questionnaire in a within-subjects design. At the start of the questionnaire, participants were told about the study, detailing what the experimental procedure would consist of, before being asked to give informed consent electronically. Participants were asked to complete a questionnaire of two parts: the first part consisting of four moral dilemmas, and the second of individual differences measures.

#### 2.1.2 Measures

##### 2.1.2.1 Moral dilemmas

Four sacrificial dilemmas involving ‘up-close-and personal’ harm were presented in random order. These ‘personal’ dilemmas were drawn from Moore, Clark, and Kane (2008) and included the classic Footbridge case, in which one can save five people from a runaway trolley only by pushing another person onto the tracks, leading to their death (see Supplementary material). Participants were first asked ‘From a moral point of view, should you [perform the ‘utilitarian’ act, e.g. push the stranger in the Footbridge case]?’ They were then asked to rate, on a scale of 1–5, the wrongness of this act. In line with prior research, both rates of explicit endorsement of the ‘utilitarian’ act and lower wrongness ratings of that act were taken as measures of a ‘utilitarian’ tendency. Participants were also asked to report how difficult the dilemma was; how confident they were about their response; and what they expected others to respond. Results for these further questions are not reported here.

##### 2.1.2.2 Business ethics

This scale was taken from Cooper and Pullig (2013) and included 6 items describing ethics violations (e.g. ‘An underpaid executive padded his expense account by about $3,000 a year’; Cronbach’s *α* = .70). For each scale item, participants were asked to rate the acceptability of the behavior described (1 = “*Never Acceptable*” *to* 7 = “*Always Acceptable*”; i.e. higher scores indicate more lenient assessment of wrongness).

##### 2.1.2.3 Psychopathy

Primary psychopathy was measured using Levenson, Kiehl, and Fitzpatrick’s primary psychopathy sub-scale (1995). This consisted of 16 items, including ‘Success is based on survival of the fittest; I am not concerned about the losers.’ (*α* = .87).

##### 2.1.2.4 Empathic concern

This scale was drawn from the Interpersonal Reactivity Index (Davis, 1980). We focused only on the Empathic Concern subscale of this index, in line with prior results tying it to reduced rates of ‘utilitarian’ judgment (Choe and Min, 2011, Crockett et al., 2010). This subscale measures sympathy and concern for others, or emotional empathy. It consists of 7 items, such as ‘When I see someone being taken advantage of, I feel kind of protective towards them’ (*α* = .75).

Participants also filled out the short Autism Quotient scale (Hoekstra et al., 2011); results for this scale are not reported here.

### 2.2 Results

Correlational analyses were conducted to investigate relationships between the individual difference measures, responses on the moral dilemmas, and ratings on the Business Ethics scale (see Table 1)^5^:

i. Overall, endorsement of ‘utilitarian’ solutions to personal moral dilemmas was associated with lower wrongness ratings of the ‘utilitarian’ action (*r* = −.68, *p* < .001). Endorsement of ‘utilitarian’ solutions was associated with primary psychopathy (*r* = .29, *p* < .001) and marginally with reduced empathic concern (*r* = −.14, *p* = .06). Lower wrongness ratings of the ‘utilitarian’ action were associated with primary psychopathy (*r* = −.32, *p* < .001) and increased wrongness ratings with empathic concern (*r* = .17, *p* = .02). A multiple regression analysis testing the effects of psychopathy and empathic concern on wrongness judgments revealed that the two factors explained 10% of the variance in perceived wrongness of the utilitarian action (*R*^2^ = .10, *F* (2, 193) = 10.61, *p* < .001), but this effect was driven solely by primary psychopathy (*β* = −.1.11, *p* < .001).
ii. Lower wrongness ratings of business ethics violations were associated with greater endorsement of ‘utilitarian’ solutions to the dilemmas (*r* = .25, *p* < .001) and lower wrongness ratings of the ‘utilitarian’ action (*r* = −.31, *p* < .001).
iii. Lower wrongness ratings of business ethics violations were associated with increased primary psychopathy (*r* = .58, *p* < .001) and reduced empathic concern (*r* = −.29, *p* < .001).
iv. The relationship between business ethics and ‘utilitarian’ answers in the dilemmas was then subjected to a first order partial correlation in order to explore the relationship controlling for psychopathy. This first order correlation was found to be significant for the perceived wrongness of the action (*r* = −.16, *p* = .03), but not with rates of categorical ‘utilitarian’ judgments. As such, it seems that while psychopathy does appear to drive at least some of the relationship between ‘utilitarian’ responses to personal dilemmas and reduced business ethics, psychopathy cannot explain this relationship fully.

**Table 1:** Correlation matrix for Study 1.

	1	2	3	4
1. ‘Utilitarian’ answers				
2. Wrongness of ‘Utilitarian’ action	−.68⁎⁎			
3. Business ethics	.25⁎⁎	−.31⁎⁎		
4. Primary psychopathy	.29⁎⁎	−.32⁎⁎	.58⁎⁎	
5. Empathic concern	−.14	.17⁎	−.29⁎⁎	−.51⁎⁎

⁎ *p* < .05*.*

⁎⁎ *p* < .01*.*

### 2.3 Discussion

In line with recent studies, we found that ‘utilitarian’ judgment was positively correlated with primary psychopathy and reduced empathic concern—traits that one would not expect to be associated with a genuine concern for the greater good. A regression analysis suggested that it was primary psychopathy rather than reduced empathic concern *per se* that drove the association with ‘utilitarian’ judgment.

Importantly, ‘utilitarian’ judgment was associated with more lenient assessment of immoral behavior in the Business Ethics measure. This association is *directly* between ‘utilitarian’ judgment and an amoral pattern of judgment, rather than, as in prior studies, only between ‘utilitarian’ judgments and reduced empathic concern or measures of antisocial personality traits. Notice, moreover, that this association was *not* fully explained by the correlation between ‘utilitarian’ judgment and psychopathy.

These results strongly suggest that so-called ‘utilitarian’ judgment is at least partly driven by a general antisocial or immoral tendency, rather than by a focused willingness to harm individuals in specific moral contexts.^6^ Note that the transgressions described in the Business Ethics measure were in the third rather than first person (that is, they involved assessing the morality of *other* people’s behavior), and did not involve serious ‘up close and personal’ harm of the kind studied by personal dilemmas (Greene et al., 2001). In fact, these transgressions often involved violations of fairness rather than of harm norms, further suggesting that the observed disposition to ‘utilitarian’ judgment reflects a broader antisocial tendency rather than a specific deficit in aversion to causing ‘personal’ harm, much less a genuine concern for the greater good.

The results of Study 1 suggest that antisocial tendencies play a significant role in driving ‘utilitarian’ judgments in sacrificial dilemmas: individuals who are disposed to make such judgments appear to be *generally* dismissive of common moral rules and norms, whether or not this promotes the greater good. One deliberate feature of Study 1, however, can also be seen as a potential limit. It might be thought that individuals with a genuine utilitarian outlook might also be more inclined to overrule conventional moral norms of the sort measured by the Business Ethics scale—norms relating, for example, to fairness or property rights. Study 1 can therefore not rule out the possibility that a tendency to ‘utilitarian’ judgment in sacrificial dilemmas might still be associated with a disposition to endorse the less conventional forms of explicit concern for the greater good that are more distinctive of a genuine utilitarian moral outlook. Study 2 was designed to address this possibility, as well as to further clarify the puzzling association between antisocial traits and moral judgments that seem responsive to utilitarian considerations about the greater good.

## 3 Study two

It may seem surprising that an antisocial tendency would manifest itself in judgments that seem to conform to a utilitarian outlook. However, an amoral, self-centered perspective and an impartial utilitarian concern for the greater good share important structural features: both use cost-benefit analyses to guide action, and both tend to dismiss many commonsense moral norms as spurious conventions that should be followed, if at all, only when this has beneficial consequences (Sidgwick, 1907). What distinguishes the egoism of the amoralist and the universal benevolence of the true utilitarian is the scope of their circle of concern: utilitarians care about the greater good, egoists only about their own good.

Study 2 was therefore designed to investigate more directly whether typical ‘utilitarian’ judgments in personal dilemmas really express greater concern for the greater good, or whether they merely express a calculating yet selfish mindset.

In order to investigate this question, we employed the following measures.

1*. Minimal altruism to distant strangers.* We more directly tested the relationship between ‘utilitarian’ judgment and the kind of impartial concern for others that is the mark of a genuine utilitarian outlook by including a scenario in which participants were told to imagine that they had received an unexpected bonus, and were then asked how much of it they would anonymously donate to a respected charity that helps people in the developing world. Whereas the Business Ethics measure employed in Study 1 asked subjects to rate the wrongness of bad behavior in the business context—a measure that assumes a broadly conventional view of morality—this measure of altruism examines moral attitudes that more directly align with classical utilitarianism. Notice, however, that donating even the entire amount of this bonus would still fall *far* short of what is arguably demanded by a genuine utilitarian ethics. Still, to the extent that utilitarian judgment reflects, however loosely, the kind of attitudes typically associated with concern for the greater good, one would surely expect a strong correlation between the two. By contrast, we predicted that there would be no such correlation, *even when controlling for antisocial tendencies*.

2*. Egoism and concern for humanity as a whole.* Philosophers distinguish three senses of egoism. According to psychological egoism, people are only *actually* motivated by their self-interest. According to rational egoism, promotion of one’s self-interest is the only *rational* course of action. According to ethical egoism, promotion of one’s own self-interest is the only *moral* course of action. Participants were asked to rate their agreement with each of these three views. To the extent that what is typically described as ‘utilitarian’ judgment expresses genuine concern for the greater good, it should be strongly negatively correlated with ethical egoism, as well as, arguably, with rational egoism. And although psychological egoism is a descriptive claim rather than a normative view, one would expect individuals with radically altruist moral beliefs to also deny the cynical view that people always act only out of selfish motives. However, given the consistent association between ‘utilitarian’ judgment and psychopathy, we predicted the contrary results.

In addition, we included the *Identification with All Humanity Scale* (IWAH), a scale that measures the extent to which individuals identify with humanity as a whole as opposed to exhibit more parochial attachment to one’s own community or country (McFarland, Webb, & Brown, 2012). Such all-encompassing, impartial concern is a core feature of classical utilitarianism (Hare, 1981). To the extent that utilitarian judgment in personal dilemmas expresses such concern for the greater good of all, one would expect a strong positive correlation between such judgment and IWAH. However, since greater IWAH is likely to be driven by greater empathic concern, we instead predicted a negative correlation between the two.

3*. ‘Utilitarian*’ *judgment and sensitivity to self-interest.* To investigate whether the seemingly ‘utilitarian’ judgments of individuals higher on psychopathy are actually especially sensitive to considerations of self-interest, we included, following Moore et al. (2008), not only personal dilemmas in which one is asked whether to sacrifice a single individual to save a group of strangers (*other-benefit* dilemmas), but also dilemmas in which, in the hypothetical scenario, this sacrifice would also benefit the participant (*self-benefit* dilemmas). To the extent that what is typically described as ‘utilitarian’ judgment really does reflect a broadly impartial, all-concerning outlook, this distinction should not make a difference to rates of such judgment. Moore et al. (2008) have already reported that rates of ‘utilitarian’ judgment are nevertheless significantly higher in self-benefit dilemmas. Here, however, we further predicted that primary psychopathy would be associated with a marked increase in ‘utilitarian’ judgment in self-benefit dilemmas, whereas, by contrast, identification with the whole of humanity would be associated with increased ‘utilitarian’ judgment in other-benefit dilemmas.

To further investigate this issue, we also included a dilemma in which, in order to save a greater number, one has the option of sacrificing *oneself*. Materials and Results for this measure are reported in the Supplementary material.

### 3.1 Method

#### 3.1.1 Participants and procedure

317 US participants were again recruited online using Amazon Mechanical Turk (MTurk), receiving $0.50 for their time. Participants were excluded from analysis (*N* = 34) if they did not complete the survey, failed an attention check or completed the survey in too short a time (<5 min) Therefore, the number of participants included in data analysis was 283 (151 female; *M_age_* = 36, *SD* = 13.07). Participants completed the survey online and all participants answered first the standard personal dilemmas (randomised for each participant), followed by the self-sacrifice dilemma, and then all other measures.

#### 3.1.2 Measures

##### 3.1.2.1 Self-beneficial and other-beneficial dilemmas

Participants were given eight personal moral dilemmas (again drawn from Moore et al. (2008); see Supplementary material). Four of these dilemmas were other-beneficial, as in Study 1, and four were self-beneficial. An example of a self-beneficial dilemma is the *Modified Crying Baby* dilemma, in which the only way to save your life and that of other civilians from getting killed by murderous enemy soldiers is to smother your crying baby. Each dilemma was followed by the same questions used in Study 1, with one addition: participants were now also asked whether they thought that they would be able to *actually* perform the ‘utilitarian’ action in real life.

##### 3.1.2.2 Hypothetical donation measure

Participants were asked to imagine that they had received a $100 bonus at work, and could anonymously choose to donate this money to charity. Participants were told that all money donated would be doubled by the employer for the charity (see Supplementary materials for full text). Participants were then asked how much of the bonus they would donate, indicating their answer on a sliding scale from $0–100.

##### 3.1.2.3 Identification with All Humanity Scale (IWAH)

This scale was taken from McFarland et al. (2012) and consisted of 9 questions, including requiring participants to rate, for people in their community, people in their country, and people all over the world, “How close do you feel to each of the following groups?” In analyzing results, the procedure advised by McFarland et al. was used, regressing the raw scores to give a more accurate representation of the variance in identification with all of humanity, whereby higher scores indicate greater identification with all of humanity (*α* = .93).

##### 3.1.2.4 Psychological, ethical, and rational egoism

In this measure, participants were given three statements designed to assess their belief in psychological, rational, and ethical egoism. Participants were required to rate how much they agreed or disagreed with each statement on a 1–7 scale (1 = *Strongly Disagree,* 7 = *Strongly Agree*). The three items were as follows: “People may sometimes appear to do things for the sake of others, but deep down, the only thing that really motivates people is their own self-interest” (Psychological Egoism); “An action isn’t rational if it doesn’t aim to promote one’s own self interest” (Rational Egoism); and “An action isn’t morally right if it doesn’t aim to promote one’s own self interest” (Ethical Egoism).

### 3.2 Results

#### 3.2.1 Individual differences and ‘Utilitarian’ judgment

Correlational analyses on the relationships between scores on the individual differences measures (see Table 2) revealed that:

i. As expected, primary psychopathy was negatively correlated with Identification With All of Humanity (IWAH) (*r* = −.40, *p* < .001), and positively associated with all three strains of egoism: psychological egoism (*r* = .36, *p* < .001), rational egoism (*r* = .58, *p* < .001), and ethical egoism (*r* = .47, *p* < .001).
ii. Pooled across all eight dilemmas, higher rates of ‘utilitarian’ judgment were again associated with primary psychopathy (*r* = .22, *p* < .001), while lower rates of ‘utilitarian’ judgment showed a trending association with IWAH (*r* = −.10, *p* = .09). When primary psychopathy was controlled for, there was no relationship between IWAH and ‘utilitarian’ judgments (*r* = −.05, *p* = .41). ‘Utilitarian’ judgments were associated with increased endorsement of rational egoism (*r* = .16, *p* < .01), though not with psychological or ethical egoism.
iii. Reduced wrongness ratings of ‘utilitarian’ actions were associated with primary psychopathy (*r* = −.17, *p* < .005), rational egoism (*r* = −.14, *p* = .02), and marginally with ethical egoism (*r* = −.12, *p* = .04), but not with IWAH (*r* = .07, *p* = .27).
iv. Participants higher on primary psychopathy were more likely to predict that they would *actually* perform the ‘utilitarian’ action (*r* = .37, *p* < .001); the reverse association was found for IWAH (*r* = −.17, *p* = .03). The relationship between primary psychopathy and the likelihood of performing the ‘utilitarian’ action was investigated next, controlling for the higher wrongness ratings and utilitarian answers associated with psychopathy. The first order partial correlation revealed that psychopathy was still significantly associated with a greater likelihood of performing the ‘utilitarian’ action, even when the judged morality of said action was controlled for (*r* = .21, *p* < .001).

**Table 2:** Correlation matrix for Study 2.

	1	2	3	4	5	6	7	8	9	10	11	12	13	14
1. Primary psychopathy														
2. Identification with humanity	−.40⁎⁎													
3. Psychological egoism	.36⁎⁎	−.26⁎⁎												
4. Rational egoism	.58⁎⁎	−.24⁎⁎	.40⁎⁎											
5. Ethical egoism	.47⁎⁎	−.22⁎⁎	.40⁎⁎	.68⁎⁎										
6. Hypothetical bonus donation	−.24⁎⁎	.27⁎⁎	−.14⁎	−.13⁎	−.07									
7. Overall endorsement of ‘Utilitarian’ action	.22⁎⁎	−.10	.05	.16⁎⁎	.07	−.12⁎								
8. Overall likelihood of performing of ‘Utilitarian’ action	.37⁎⁎	−.13⁎	.17⁎⁎	.24⁎⁎	.16⁎⁎	−.05	.58⁎⁎							
9. Overall wrongness of the ‘Utilitarian’ action	−.17⁎⁎	.07	.01	−.14⁎	−.12⁎	.02	−.58⁎⁎	−.49⁎⁎						
10. Self-beneficial dilemmas endorsement of ‘Utilitarian’ action	.24⁎⁎	−.10	.09	.19⁎⁎	.11	−.18⁎⁎	.92⁎⁎	.53⁎⁎	−.51⁎⁎					
11. Self-beneficial likelihood of performing of ‘Utilitarian’ action	.41⁎⁎	−.14⁎	.20⁎⁎	.26⁎⁎	.18⁎⁎	−.11	.55⁎⁎	.93⁎⁎	−.44⁎⁎	.57⁎⁎				
12. Self-beneficial ‘Wrongness’ of the ‘Utilitarian’ action	−.19⁎⁎	.07	.02	−.12⁎	−.12⁎	.03	−.53⁎⁎	−.46⁎⁎	.95⁎⁎	−.53⁎⁎	−.47⁎⁎			
13. Other-beneficial endorsement of ‘Utilitarian’ action	.16⁎⁎	−.09	.01	.09	.01	−.04	.92⁎⁎	.54⁎⁎	−.55⁎⁎	.69⁎⁎	.43⁎⁎	−.46⁎⁎		
14. Other-beneficial likelihood of performing of ‘Utilitarian’ action	.28⁎⁎	−.10	.12⁎	.18⁎⁎	.12⁎	.01	.53⁎⁎	.93⁎⁎	−.47⁎⁎	.41⁎⁎	.72⁎⁎	−.38⁎⁎	.57⁎⁎	
15. Other-beneficial ‘Wrongness’ of the ‘Utilitarian’ action	−.13⁎	.05	.01	−.15⁎	−.11	.01	−.56⁎⁎	−.47⁎⁎	.94⁎⁎	−.44⁎⁎	−.36⁎⁎	.78⁎⁎	−.59⁎⁎	−.51⁎⁎

⁎ *p* < .05.

⁎⁎ *p* < .01.

#### 3.2.2 Other- vs. self-beneficial dilemmas

Concordant with previous research (Moore et al., 2008), analysis of the relationship between other- and self-beneficial dilemmas revealed (see Table 3):

i. Greater endorsement of the ‘utilitarian’ option in the self-beneficial case (*M* = 1.50) compared to the other-beneficial case (*M* = 1.40), *t*(283) = 6.29, *p* < .001.
ii. Lower wrongness ratings in the self-beneficial cases (*M* = 3.67) compared to the other-beneficial cases (*M* = 3.75), *t*(285) = −2.35, *p* = .02.
iii. A greater likelihood of performing the ‘utilitarian’ action was reported in the self-beneficial cases (*M* = 3.12) compared to the other-beneficial cases (*M* = 2.85), *t*(283) = 4.44, *p* < .001.

**Table 3:** Comparison of self beneficial and other beneficial dilemmas in Study 2.

	Self-beneficial		Other-beneficial		*t-Test*
	*M*	*SD*	*M*	*SD*	
Endorsement of ‘Utilitarian’ action	1.50	0.35	1.40	0.32	*t*(283) = 6.29, *p* < .001
Wrongness of ‘Utilitarian’ action	3.67	0.88	3.75	0.86	*t*(285) = −2.35, *p* = .02
Likelihood of performing the ‘Utilitarian’ action	3.12	1.36	2.85	1.37	*t*(283) = 4.44, *p* < .001

Correlational analyses were then conducted looking at the self-beneficial and other-beneficial dilemmas in isolation (see Table 2). These analyses showed that:

i. Primary psychopathy was significantly correlated with ‘utilitarian’ answers in both the other-beneficial (*r* = .16, *p* < .01) and self-beneficial dilemmas (*r* = .24, *p* < .001), and a greater likelihood of performing the ‘utilitarian’ action in both the self-beneficial (*r* = .41 *p* < .001) and other-beneficial cases (*r* = .28 *p* < .001). The relationship between primary psychopathy and the likelihood of performing the self-beneficial or other-beneficial ‘utilitarian’ actions was also again investigated controlling for wrongness ratings and endorsement of the ‘utilitarian’ action. The first order partial correlations revealed that psychopathy was still significantly associated with a greater likelihood of performing both the self-beneficial ‘utilitarian’ action (*r* = .34, *p* < .001) and the other-beneficial ‘utilitarian’ action (*r* = .22, *p* < .001).
ii. Identification With All of Humanity (IWAH) was not correlated with ‘utilitarian’ judgments in either the other-beneficial (*r* = −.09, *p* = .14) or self-beneficial dilemmas (*r* = −.10, *p* = .10), nor was it correlated with wrongness ratings in either self-beneficial dilemmas (*r* = .07, *p* = .24) or other-beneficial dilemmas (*r* = .05, *p* = .37). Interestingly, however, IWAH was associated with reduced likelihood of predicting that one would actually perform the ‘utilitarian’ action in the self-beneficial (*r* = −.14, *p* = .02) but not other-beneficial cases (*r* = −.10 *p* = .08).

Next, an ANOVA was conducted to investigate whether there was a significant interaction effect between primary psychopathy and scores on the two types of dilemma: were individuals high on primary psychopathy more likely to perform the ‘utilitarian’ action in the self-beneficial case? Results from a mixed design ANOVA with bonferroni correction (Within-Subjects: self-beneficial dilemmas vs, other-beneficial dilemmas; Between-Subjects: primary psychopathy using median split) showed a significant interaction effect of primary psychopathy and dilemma type on how likely the participants were to predict that they would *actually* perform the ‘utilitarian’ action, *F* (1, 281) = 5.59, *p* = .02. Those higher on primary psychopathy were significantly more likely than those low in psychopathy to perform both the self-beneficial and other-beneficial ‘utilitarian’ action. For individuals low in primary psychopathy, however, pairwise comparisons revealed that there was no difference in likelihood of actually performing the self- or other-beneficial act (*p* = .19). Subjects higher on psychopathy reported being significantly more likely to perform the ‘utilitarian’ action in the self-beneficial cases (*p* < .001).

Further results from the same mixed design ANOVA with bonferroni correction (Within-subjects: self-beneficial dilemmas vs. other-beneficial dilemmas; Between-subjects: primary psychopathy using median split) on different dependent variables showed no significant interaction effect of primary psychopathy and dilemma type on how wrong the ‘utilitarian’ action was judged to be, *F* (1, 281) = 3.05, *p* = .08, or on whether the participant endorsed the utilitarian option, *F* (1, 281) = 1.90, *p* = .17.

#### 3.2.3 Minimal altruism to distant strangers

Next, correlational analyses were conducted to explore the relationship between donations in the hypothetical donation vignette and other variables, revealing that:

i. As expected, primary psychopathy was associated with smaller amounts of money donated (*r* = −.24, *p* < .001), while IWAH predicted more money donated (*r* = .27, *p* < .001) (see Table 2).
ii. Greater rates of ‘utilitarian’ judgment were negatively correlated with money donated in the hypothetical donation: ‘utilitarians’ donated less money than those who did not endorse the ‘utilitarian’ response in the moral dilemmas (*r* = −.12, *p* = .04). Importantly, this lack of a positive relationship between ‘utilitarian’ judgment overall and more money donated in the hypothetical donation measure held even when controlling for primary psychopathy through a partial correlation technique (*r* = −.07, *p* = .23).

### 3.3 Discussion

Study 2 directly investigated the relationship between ‘utilitarian’ judgment in sacrificial dilemmas and a range of markers of impartial concern for the greater good and its contrary, exclusive egoist concern for one’s own self. Some of these markers involved judgments and attitudes that are either paradigmatic of a genuine utilitarian outlook (e.g. greater willingness to help distant others in need, and greater identification with humanity as a whole) or directly opposed to such an outlook (e.g. endorsement of explicit egoist views). Others were internal to the context of a sacrificial dilemma (greater willingness to sacrifice others when this is in one’s own benefit).

We considered the relationship between ‘utilitarian’ judgment and these markers both in general as well as when subclinical psychopathic tendencies were controlled for. Across the board, a tendency toward ‘utilitarian’ judgment was associated with *lower* rates of attitudes expressive of an impartial concern for the greater good—reduced rates of hypothetical donation and identification with the whole of humanity—and *increased* endorsement of rational egoism (though not of psychological or ethical egoism). When psychopathic tendencies were controlled for, *no* association was found between ‘utilitarian’ judgment and these other measures.

These findings offer strong further evidence in support of our hypothesis that, on the whole, so-called ‘utilitarian’ judgment is often driven, not by concern for the greater good, but by a calculating, egoist, and broadly amoral outlook. Importantly, however, even when we controlled for the antisocial component in ‘utilitarian’ judgment, an association with broader concern for the greater good did not emerge.

Further evidence for this hypothesis emerged *within* the context of sacrificial dilemmas. We found that although individuals with sub-clinical psychopathic tendencies may appear, in this unusual context, to be making judgments that aim at maximizing the good, these judgments are in fact highly sensitive to considerations of self-interest—considerations that should be out of place if one were genuinely aiming to promote the greater good from an impartial utilitarian standpoint.

Interestingly, individuals who were higher on psychopathy were also significantly more likely to report that they would be able to *actually commit* the ‘utilitarian’ act compared to participants who scored low on psychopathy; this difference was significantly stronger in the self-benefit dilemmas compared to the other-benefit ones. By contrast, higher identification with the whole of humanity was associated with reduced likelihood of actually performing the ‘utilitarian’ action, but only in the self-beneficial category.

The extent to which an individual identifies with the whole of humanity is best seen as an affective disposition rather than a moral view—although this is an affective disposition that is strongly linked to an impartial moral outlook, and central to many classical and contemporary utilitarian views (e.g. Hare, 1981). In line with this, greater identification with the whole of humanity was also associated with donation of more of an unexpected bonus to people in need in the developing world. It was also, as could be expected, negatively correlated with psychopathy and egoist views. Yet there was a trend toward a negative correlation between identification with the whole of humanity and endorsement of ‘utilitarian’ solutions in sacrificial dilemmas.

## 4 Study 3

Study 2 provided additional evidence that ‘utilitarian’ judgment is associated with attitudes that are contrary to genuine utilitarian concern for the greater good. Study 3 aimed to further investigate this question by expanding on Study 2 in several respects. Instead of considering the relationship between ‘utilitarian’ judgment and paradigmatic utilitarian attitudes (identification with the whole of humanity) and hypothetical behavior (donation to help people in developing countries), we considered its relationship to a wide range of explicit moral judgments that are characteristic of a genuine utilitarian moral outlook, when it is applied to real world questions rather than to unusual hypothetical scenarios.

Utilitarians hold, among other things, the following: that we should not give moral priority to people in need from our own country over people in greater need from other countries; that well-off individuals in Western countries therefore ought to give some of their money to help people in need in poor countries; and that they should also be willing to make significant sacrifices now to prevent environmental damage that would cause great harm to future generations. These views are familiar form the work of Peter Singer, the most famous living utilitarian (Singer, 1972, Singer, 1979), but also endorsed by many other leading utilitarians and consequentialists (see e.g. Glover, 1977, Kagan, 1989, Rachels, 1996). These characteristic utilitarian judgments all involve impartially taking into account the good of all rather than privileging some narrower group of individuals—let alone privileging one’s own selfish interests.

To the extent that a tendency to ‘utilitarian’ judgment in sacrificial dilemmas in fact reflects greater concern for the greater good, we would expect such a tendency to be positively associated with these characteristic real-world utilitarian judgments. By contrast, we again predicted that ‘utilitarian’ judgment would be negatively correlated with these views that express positive impartial concern for the greater good. We further predicted that no relation would be observed between ‘utilitarian’ judgment and such real-life utilitarian views once psychopathy is controlled for.

### 4.1 Method

#### 4.1.1 Participants and procedure

233 American participants were again recruited online using Amazon MTurk and were paid $0.50 for their time. Participants were excluded from analysis (*N* = 43) if they did not complete the survey, failed an attention check or completed the survey in too short a time (<250 s). Therefore, the total number of participants included in data analysis was 190 (94 females; *M_age_* = 36, *SD* = 13.51).

Participants completed four personal moral dilemmas (the ‘other-beneficial’ dilemmas used in Study 2) and the hypothetical donation measure used in Study 2. They also filled in the primary psychopathy part of Levenson’s *Psychopathy Self Report Scale*, and reported demographic information. In addition, participants completed a short questionnaire tapping ‘real-world’ utilitarian attitudes and ‘real-world’ harm, described below. To avoid potential order effects, questions were presented in a semi-random order.

#### 4.1.2 Measures

##### 4.1.2.1 Real-world utilitarianism

Participants completed a set of four questions adapted by the present researchers from the writings of major contemporary utilitarian authors to obtain a measure of characteristic real-world utilitarian judgments. Items included questions on the extent to which participants think that well-off people in the West have moral obligations to help poor people in developing countries; obligations to give priority to people in great need in very poor foreign countries over people in lesser need in one’s own country; obligations to make sacrifices for the sake of future generations; and the wrongness of failing to donate money to help children in need in poor countries (before this last question, participants were first asked whether it is wrong not to save a drowning child at little cost to oneself, following Singer, 1972; see Supplementary materials for full details on questions asked). Scores on these items were aggregated to form a measure of real-world utilitarian beliefs (*α* = .64), and all items were significantly positively inter-correlated at the *p* < .001 level (see Table 4).

**Table 4:** Real life utilitarianism items inter-correlation for Study 3.

	1	2	3
1. Wrongness of not helping children in poor countries			
2. Obligations of wealthy in the west	.44^∗∗^		
3. Helping a foreign country over own country	.29^∗∗^	.43^∗∗^	
4. Sacrifices to prevent climate change	.23^∗∗^	.31^∗∗^	.33^∗∗^

Note: *p*s < .001.

##### 4.1.2.2 Real-world harm

In addition, participants completed four further questions about the moral permissibility of causing significant harm in real-life contexts (abortion, experimentation in animals, eating meat, and torture). These were included to investigate whether ‘utilitarian’ judgment in personal dilemmas is associated with greater willingness to endorse harm in real-life contexts, even when an explicit utilitarian rationale for that harm is not provided. These items were not collated into a scale due to low internal reliability (*α* = .07), and were therefore analyzed separately.

### 4.2 Results

Correlational analyses were conducted to explore the relationship between primary psychopathy, responses to the personal moral dilemmas, and the new measure of characteristic real-world utilitarian judgment (see Table 5), revealing:

i. Reduced wrongness ratings of ‘utilitarian’ responses in the moral dilemmas were not significantly correlated with real-world utilitarian beliefs (*r* = −.03, *p* = .72). This lack of a relationship held even when controlling for primary psychopathy, yielding a non-significant partial correlation (*r* = .02, *p* = .81). Real-life utilitarian beliefs were associated with increased hypothetical donations (*r* = .49, *p* < .001) and thinking that both eating meat (*r* = .32, *p* < .001) and torture (*r* = −.23, *p* < .005) are more wrong, and that painful animal experimentation is less acceptable (*r* = .28, *p* < .005). By contrast, ‘utilitarian’ judgments in the personal dilemmas were associated with finding painful animal experimentation more acceptable (*r* = .28, *p* < .001) but abortion more wrong (*r* = .22, *p* < .005).
ii. Surprisingly, in this study primary psychopathy was not associated with lower wrongness ratings of ‘utilitarian’ actions in the moral dilemmas (*r* = −.05, *p* = .53). Psychopathy was again associated with reduced donations in the hypothetical donation (*r* = .17, *p* = .02), as well as with thinking that real-world harm is more acceptable, for eating meat (*r* = −.15, *p* = .03), animal experimentation (*r* = −.15, *p* = .04) and abortion (*r* = −.21, *p* = <.005). By contrast, psychopathy was also associated with reduced endorsement of real-life utilitarian judgments (*r* −.17, *p* = .04). That is, individuals relatively higher in psychopathy found harm more morally acceptable in real-life moral contexts, yet were *less* utilitarian with regard to real life issues.

**Table 5:** Correlation matrix for Study 3.

	1	2	3	4	5	6	7	8
1. Primary psychopathy								
2. Personal sacrificial dilemmas endorsement	−.05							
3. Personal sacrificial dilemmas wrongness	−.05	.59⁎⁎						
4. Real life utilitarianism	−.17⁎	.01	−.03					
5. Eating meat	−.15	.05	.11	.32⁎⁎				
6. Animal experimentation	−.15⁎	−.08	−.22⁎⁎	.28⁎⁎	.30⁎⁎			
7. Abortion	−.21⁎	−.13	−.22⁎⁎	−.02	−.05	−.04		
8. Torture	.12	.04	−07	−.23⁎⁎	−.27⁎⁎	−.17⁎	.15⁎	
9. Hypothetical donation	−.17⁎	.06	−.07	.49⁎⁎	.21⁎⁎	.14⁎	.09	−.10

⁎ *p* < .05.

⁎⁎ *p* < .01.

### 4.3 Discussion

In this study, we directly investigated the relationship between ‘utilitarian’ judgment in sacrificial dilemmas and some of the moral judgments most closely associated with a utilitarian outlook when it is applied to the real world. We found no relationship between these two sets of moral judgments: individuals who were more willing to endorse sacrificing one person to save a greater number did not also exhibit more impartial moral views in contexts that involve impartial altruism and potential self-sacrifice—views that are the very heart of a utilitarian outlook. These results provide yet further support for our hypothesis that willingness to endorse personal harm in hypothetical dilemmas is not expressive of impartial concern for the greater good.

## 5 Study 4

In Study 3 we examined a range of real life moral views that are characteristic of a utilitarian ethical outlook—for example, the view that we should donate significant amounts of our income to charities that save lives. Such moral views, however, depend on (plausible) empirical assumptions that were not always made explicit in Study 3, and that some individuals may not share—i.e., someone may have strong utilitarian leanings yet also believe that aid is a highly ineffective way of helping people in need. In order to address this issue, in Study 4 we constructed a series of new vignettes that spell out, in an explicit manner, that the more impartial choice is also the one that will lead to a greater overall good—for example, saving the lives of several children in a distant country as opposed to one in one’s own country; helping disaster victims as opposed to buying a car or mobile phone; and saving an important peace-maker as opposed to saving one’s own mother. These ‘greater good’ vignettes thus directly pit an explicit utilitarian action promoting the greater good against a narrower, more partial moral view that allows us to give priority to self, family, and country. Moreover, in this study the standard sacrificial dilemmas were compared to similarly presented vignettes, addressing the possibility that prior results were partly influenced by differences in the way moral questions were presented across stimuli. In line with our prior findings, we predicted that ‘utilitarian’ judgments in sacrificial dilemmas would be negatively correlated with genuinely utilitarian judgments in these new vignettes, and that this correlation would be driven by the antisocial dimension of sacrificial ‘utilitarian’ judgments. We again further predicted that there would be no correlation between these two sets of judgments once this antisocial dimension was controlled for.

Study 4 included one additional measure. The new vignettes, as well as the measures employed in the prior studies, assessed concern for the greater good only at an abstract or hypothetical level—asking in Study 2, for example, how much of a hypothetical bonus participants would be willing to donate to charity. In Study 4 we added a measure of actual altruistic behavior aiming to promote the greater good, by offering participants the option of donating part of an actual small sum to a recognized charity that has been shown to be effective in saving lives in developing countries. We predicted that such donation would be negatively correlated with more ‘utilitarian’ responses to sacrificial dilemmas while positively correlated with endorsement of characteristic utilitarian views in the new ‘greater good’ vignettes.

### 5.1 Method

#### 5.1.1 Participants and procedure

253 American participants were again recruited online using Amazon MTurk and were paid $0.50 for their time. Participants were again excluded from analysis (*N* = 21) if they failed an attention check or completed the survey in too short a time (<250 s). The total number of participants included in data analysis was 232 (117 females; *M_age_* = 38, *SD* = 13.41). To avoid potential order effects, questions were presented in a random order.

#### 5.1.2 Measures

As in previous studies, participants completed the four personal moral dilemmas (the personal ‘other-beneficial’ dilemmas used in Studies 2 and 3), filled in the measure of primary psychopathy, and reported demographic information. In addition, participants completed 2 new measures:

##### 5.1.2.1 ‘Greater Good’ dilemmas

Participants completed seven new ‘greater good’ vignettes (see Supplementary material) tapping the impartial concern for the greater good that characterizes genuine utilitarianism. Each such vignette presented a possible choice (e.g. donating to charity that would save one life in one’s own country vs. donating to a charity that would save a greater number in a foreign country), and participants were then asked to rate the wrongness of failing to choose the more utilitarian option. Note that in contrast to the classical personal dilemmas, in these new ‘greater good’ dilemmas higher wrongness ratings indicated a more utilitarian view (*α* = .77).

##### 5.1.2.2 Altruistic donation

As a behavioral measure of impartial altruism, participants were given the opportunity to actually donate to charity part of a bonus fee that they received for taking part in the study. In addition to a participation payment of $0.50, participants were offered “a bonus fee of up to $1.00, of which you can choose how much to keep and how much to donate to one out of several of the leading charities dedicated to eliminating serious disease and poverty in the third world, according to the *Giving What You Can Research Centre*. According to this respected Research Centre, even small donations to these charities will actually contribute to saving lives in developing countries.”

### 5.2 Results

#### 5.2.1 Sacrificial vs. greater good dilemmas

Correlational analyses were conducted to explore the relationship between perceived wrongness in the sacrificial personal dilemmas, perceived wrongness in the new ‘greater good’ dilemmas, primary psychopathy, and actual altruistic donations (see Table 6):

i. As in the previous studies, psychopathy was associated with reduced wrongness ratings of ‘utilitarian’ actions in the personal dilemmas (*r* = −.32, *p* < .001), but was not associated with rates of genuinely utilitarian judgment in the ‘greater good’ dilemmas (*r* = −.02, *p* = .73).
ii. There was no relationship between perceived wrongness in personal dilemmas and in the ‘greater good’ dilemmas (*r* = −.04, *p* = .53): that is, people who were more ‘utilitarian’ in the personal dilemmas were not more likely to be more truly utilitarian in the other dilemmas, and vice versa. This lack of relationship held even when controlling for primary psychopathy in a partial second order correlation (*r* = −.07, *p* = .32).
iii. Contrary to expectations, the amount donated in the charity measure was not significantly associated with ‘utilitarian’ judgments in the personal dilemmas (*r* = .10, *p* = .15) or in the new ‘greater good’ dilemmas (*r* = −.07, *p* = .30), or with psychopathy (*r* = .02, *p* = .78). The lack of relationship between donations and responses in the dilemmas again held when controlling for psychopathy: for both the personal dilemmas (*r* = .11, *p* = .10), and ‘greater good’ dilemmas (*r* = −.07, *p* = .30).

**Table 6:** Correlation matrix for Study 4.

	1	2	3
1. Sacrificial personal dilemmas			
2. Greater good dilemmas	−.03		
3. Psychopathy	−.33^∗∗^	.11	
4. Charity donation	−.10	.06	−.04

Note: *p*s < .001.

#### 5.2.2 Personal harm, self sacrifice and impartiality

We next conducted a factor analysis to explore the internal relationship between the 4 personal and 7 ‘greater good’ dilemmas. First, the factorability of the 11 dilemmas was examined. The KMO measure of sampling adequacy was .75, above the recommended value of .6, and Bartlett’s test of sphericity was significant (*χ*^2^ (55) = 535.69, *p* < .001). Given these indicators, factor analysis was conducted with all 11 items. Principle components analysis using direct oblimin rotation was used, and three significant factors were extracted: the first factor (*eigenvalue* = 2.67) explained 24% of the variance, the second factor explained 22% (*eigenvalue* = 2.37), and the third factor explained 11% (*eigenvalue* = 1.17). The analysis revealed that the four personal dilemmas loaded onto the first factor, with all of the ‘greater good’ dilemmas loading onto the second and third factors (see Table 7). This loading pattern indicated that the personal moral dilemmas used in the previous studies loaded well together (henceforth the *personal harm* factor). The second factor consisted of the new ‘greater good’ dilemmas concerning a strong component of self-sacrifice (henceforth the *impartiality vs. self-interest* factor). Finally, the remaining dilemmas concerning moral impartiality with respect to those to whom one stands in special relationships (privileging the greater good over one’s family or country) loaded onto the third factor (henceforth the *impartiality vs. privileged others* factor). The results of this factor analysis thus further support our hypothesis that ‘utilitarian’ judgment in personal dilemmas is distinct from paradigmatic utilitarian judgment in contexts relating to altruistic action involving self-sacrifice or an impartial outlook.

**Table 7:** Factor loadings for dilemmas in Study 4.

	Factor loadings		
	Personal dilemmas	Greater good dilemmas	
	Personal harm	Self sacrifice	Impartiality
How wrong would it be for Charlie to push this stranger onto the tracks to save the five workmen?	**−0.68**	0.46	
How wrong would it be for Helen to crash the other rider to avoid the deaths of the riders behind her?	**−0.65**	0.42	0.13
How wrong would it be to for Dorothy to kill one of these people with a deadly injection in order to identify a vaccine that will save thousands of lives?	**−0.61**	0.44	
How wrong would it be to push the un-harnessed crewmember off the container to his death to save the people below?	**−0.53**	0.53	0.18
How wrong would it be for Veronica to continue to enjoy her comfortable life instead of giving away large sums of money to charity?	0.38	**0.63**	−0.37
How wrong would it be for John to throw the envelope away and buy a new mobile phone?	0.42	**0.59**	−0.37
How wrong would it be for Benjamin to go on and buy the car, rather than donate any of the money?	0.43	**0.54**	−0.36
How wrong would it be for Kathleen to continue to eat meat?	0.16	**0.50**	0.21
How wrong would it be for Albert to save his mother?	0.50	0.16	**0.52**
How wrong would it be for Janet to visit her mother instead of going on to volunteer?	0.47	0.34	**0.49**
How wrong would it be for Mark to donate to the charity in his own country?	0.37	0.30	**0.39**

Note: Primary loadings are indicated by bold font. The full text for each dilemma can be seen in the Supplementary materials.

Next, we again explored how the three factors of *personal harm*, *impartiality vs. self-interest*, and *impartiality vs. privileged others* were related to each other, and to psychopathy and charitable donation (see Table 8):

i. Psychopathy was associated with greater endorsement of the ‘utilitarian’ action in *personal harm* dilemmas (*r* = −.32, *p* < .001), and greater endorsement of the typical utilitarian options in the *impartiality vs. privileged others* dilemmas (*r* = .19, *p* = .004). However, psychopathy was also significantly negatively correlated with judgments in the *impartiality vs. self-interest* dilemmas, such that individuals relatively higher in psychopathy were less truly utilitarian in dilemmas requiring self-sacrifice for the greater good (*r* = .15, *p* = .02).
ii. Genuinely utilitarian judgment in the *impartiality vs. privileged others* dilemmas was significantly related to ‘utilitarian’ judgment in the *personal harm* dilemmas (*r* = −.16, *p* = .02), such that believing it was less wrong to sacrifice one to save a greater number (e.g. by pushing a man off a footbridge) was also associated with thinking that being impartial in moral decisions (e.g. by saving one’s mother over a peace negotiator) was more acceptable. When controlling for psychopathy in a partial correlation, however, this correlation ceased to be significant (*r* = −.11, *p* = .09), suggesting that this relationship is driven primarily by psychopathy, rather than by a deeper connection between the two factors.
iii. Genuinely utilitarian judgment in the *impartiality vs. self interest* dilemmas was not associated with ‘utilitarian’ judgment in the *personal harm* dilemmas (*r* = .04, *p* = .51), and this held even controlling for psychopathy (*r* = −.02, *p* = .79).
iv. Genuinely utilitarian judgments in the *impartiality vs. self-interest* and the *impartiality vs. privileged others* dilemmas were significantly correlated (*r* = .35, *p* < .001), such that belief that we should not privilege those who are closer to us in our moral decisions was also associated with believing that we should make self-sacrifices for the greater good. This relationship held when controlling for psychopathy (*r* = .39*, p* < .001).

**Table 8:** Component correlation matrix for Study 4.

	1	2	3	4
1. Personal harm				
2. Self-sacrifice	.04			
3. Impartiality	−.12	.50⁎⁎		
4. Psychopathy	−.33⁎⁎	−.04	.28⁎⁎	
5. Charity donation	−.10	.08	.02	−.04

^∗^ *p* < .05.

⁎⁎ *p* < .01.

### 5.3 Discussion

As in Study 3, we found no association between supposedly ‘utilitarian’ judgments in sacrificial personal dilemmas and characteristic utilitarian judgments relating to assistance to distant people in need, self-sacrifice and impartiality, even when the utilitarian justification for these judgments was made explicit and unequivocal and when the moral scenarios were presented in the same manner as classical sacrificial dilemmas. Again, this lack of association remained even when we controlled for the antisocial element in ‘utilitarian’ judgment.

A factor analysis confirmed the division between sacrificial dilemmas and the ‘greater good’ dilemmas. It also revealed a further distinction, between those vignettes that involved self-sacrifice to assist distant strangers in need, and those that involved a more explicit choice between partiality to family and country and promotion of the greater good. This division is not surprising since it is plausible that self-interest and partial commitments to family and community are independent forces opposing complete moral impartiality. Indeed, in line with this, we found that while individuals higher on psychopathy were more inclined to discount moral obligations to make sacrifices for the sake of strangers, they were also less inclined to put family and country before the greater good, presumably reflecting weaker personal attachments.

To our surprise, there was no association between actual charitable donation rates and either the greater good vignettes or the classical sacrificial dilemmas. Indeed there was also no negative association between donation rates and psychopathy. These results are puzzling, given that in Studies 2 and 3 psychopathy was negatively associated with hypothetical donation, and that several of the greater good vignettes explicitly referred to similar acts of donation. This discrepancy might reflect a gap between concern for the greater good as a moral view and as a motivational state leading to actual behavior. Another possibility is that the much higher donation figures mentioned in some of the vignettes made the very small amount participants could actually donate seem too small to make a real difference. In addition, since donation rates were relatively small (*M* = $0.36; 41% donated nothing), a floor effect might also explain the lack of association with any of the other measures (see Table 9).

**Table 9:** Means and SDs of measures across all studies.

	*M*	*SD*
*Study 1*		
‘Utilitarian’ answers	1.54	0.36
Wrongness of ‘Utilitarian’ action	3.52	1.07
Empathic concern	3.55	0.71
Primary psychopathy	2.28	0.30
Business ethics	3.61	1.16

*Study 2*		
Identification with humanity	−0.02	6.10
Primary psychopathy	1.80	0.51
Hypothetical bonus donation	36.00	29.53
Psychological egoism	4.07	1.70
Rational egoism	2.61	1.43
Ethical egoism	2.32	1.35
Overall endorsement of ‘Utilitarian’ action	1.45	0.30
Overall likelihood of performing of ‘Utilitarian’ action	2.98	1.26
Overall wrongness of the ‘Utilitarian’ action	3.71	0.82
Self-beneficial dilemmas endorsement of ‘Utilitarian’ action	1.50	0.34
Self-beneficial likelihood of performing of ‘Utilitarian’ action	3.12	1.36
Self-beneficial wrongness of the ‘Utilitarian’ action	3.67	0.88
Other-beneficial endorsement of ‘Utilitarian’ action	1.40	0.32
Other-beneficial likelihood of performing of ‘Utilitarian’ action	2.85	1.37
Other-beneficial wrongness of the ‘Utilitarian’ action	3.75	0.86

*Study 3*		
Primary psychopathy	2.22	0.31
Personal sacrificial dilemmas endorsement	1.38	0.34
Personal sacrificial dilemmas wrongness	2.63	1.42
Real life utilitarianism	3.05	0.69
Eating meat	2.13	1.66
Animal experimentation	4.89	1.77
Abortion	3.07	2.08
Torture	1.68	0.61
Hypothetical donation	29.73	27.20

*Study 4*		
Primary psychopathy	1.80	0.45
Sacrificial personal dilemmas	4.95	1.44
Greater good dilemmas	1.86	0.77
Charity donation	32.98	35.86

## 6 General discussion

A great deal of recent research has focused on hypothetical moral dilemmas in which one person needs to be sacrificed in order to save the lives of a greater number. It is widely assumed that these far-fetched sacrificial scenarios can shed new light on the fundamental opposition between utilitarian and non-utilitarian approaches to ethics (Greene, 2008, Greene et al., 2004, Singer, 2005).

However, such sacrificial dilemmas are merely one context in which utilitarian considerations happen to conflict with opposing moral views (Kahane & Shackel, 2010). To the extent that ‘utilitarian’ judgments in sacrificial dilemmas express concern for the greater good—that is, the utilitarian aim of impartially maximizing aggregate welfare—then we would expect such judgments to be associated with judgments and attitudes that clearly express such concern in other moral contexts.

The set of studies presented here directly tested this prediction by investigating the relationship between so-called ‘utilitarian’ judgments in classical sacrificial dilemmas and a genuine impartial concern for the greater good. Across four experiments employing a wide range of measures and investigations of attitudes, behavior and moral judgments, we repeatedly found that this prediction was not borne out: a tendency to endorse the violent sacrifice of one person in order to save a greater number was *not* (or even negatively) associated with paradigmatic markers of utilitarian concern for the greater good. These included identification with humanity as a whole; donation to charities that help people in need in other countries; judgments about our moral obligations to help children in need in developing countries, and to prevent animal suffering and harm to future generations; and an impartial approach to morality that does not privilege the interests of oneself, one’s family, or one’s country over the greater good. This lack of association remained even when the utilitarian justification for such views was made explicit and unequivocal. By contrast, many (though not all) of these markers of concern for the greater good were inter-correlated.

In fact, responses designated as ‘utilitarian’ in the current literature were strongly associated with traits, attitudes and moral judgments (primary psychopathy, rational egoism, and a lenient attitude toward clear moral transgressions) that are *diametrically opposed* to the impartial concern for the greater good that is at the heart of utilitarian ethics. While prior studies have already associated ‘utilitarian’ judgment with antisocial traits (Bartels and Pizarro, 2011, Glenn et al., 2010, Koenigs et al., 2012, Wiech et al., 2013), here we show that such judgments are also tied to explicit amoral and self-centered judgments. Moreover, while these further associations were largely driven by antisocial tendencies, some (such as the more lenient attitude toward clear moral transgressions) were present even when we controlled for these antisocial traits.

We wish to emphasize, however, that our main result—the lack of association between ‘utilitarian’ judgment in sacrificial dilemmas and markers of concern for the greater good in other contexts—remained *even when we controlled for the antisocial component of ‘utilitarian*’ *judgment*. Thus, even if some individuals arrive at more ‘utilitarian’ conclusions in sacrificial dilemmas, not because of indifference to harming others but by deliberative effort (Conway and Gawronski, 2013, Gleichgerrcht and Young, 2013, Wiech et al., 2013) such a supposedly ‘utilitarian’ tendency is *still* not associated with paradigmatic utilitarian judgments in other moral contexts.

Several limitations of the present study need to be highlighted. First, one of our key results is a lack of correlation between ‘utilitarian’ judgments in sacrificial dilemmas and markers of impartial concern for the greater good, and it might be objected that this null result could be due to lack of statistical power. However, consistently with prior studies (Kahane et al., 2012), the present study failed to find such an association across four experiments employing a wide range of measures, with large sample sizes, while repeatedly finding associations between ‘utilitarian’ judgment and antisocial and self-centered traits, judgments and attitudes. Thus, while we cannot rule out the possibility that such an association could emerge in future studies using an even larger number of subjects or different measures, we submit that, in light of the present results, a robust association between ‘utilitarian’ judgment and genuine concern for the greater good seems extremely unlikely.

A second potential limitation is that the present study does not directly investigate the proximal causal antecedents of ‘utilitarian’ judgment in sacrificial dilemmas, and the results reported here are correlational. It might thus be objected that while our results suggest that individuals with ‘utilitarian’ tendencies in sacrificial dilemmas do not exhibit similar tendencies in other moral contexts, these findings cannot rule out that ‘utilitarian’ judgments *within* the context of sacrificial dilemmas are nevertheless driven by the utilitarian aim of impartially maximizing the greater good. In response, let us highlight first that the common reference in the literature to a utilitarian bias or to the processes underlying utilitarian decision-making suggests a generality that is incompatible with our results. At most, such claims could relate to biases or processes underlying such judgment in a very specific (and unusual) context. Second, while some of our results relate to markers of impartial concern for the greater good in moral contexts that are different from that of sacrificial dilemmas, others investigate such markers *within* this context. As we reported in Study 2, a tendency to ‘utilitarian’ judgment may in fact be strongly tied to considerations of self-interest (see also Moore et al., 2008). Several prior studies similarly found that rates of ‘utilitarian’ judgment are strongly influenced by whether they involve sacrificing (or saving) foreigners vs. compatriots (Swann, Gómez, Dovidio, Hart, & Jetten, 2010), strangers vs. family members (Petrinovich, O’Neill, & Jorgensen, 1993), and black people vs. white people (Uhlmann, Pizarro, Tannenbaum, & Ditto, 2009)—let alone animals vs. humans (Petrinovich, O’Neill, & Jorgensen, 1993). There is thus considerable evidence that judgments standardly designated as ‘utilitarian’ do not in fact aim to impartially maximize the greater good. Finally, as we shall outline below, there is an alternative, simpler account of what drives supposedly ‘utilitarian’ judgment, an account that avoids implausibly attributing to ordinary folk radical moral aims drawn from philosophy.

### 6.1 What really drives so-called ‘utilitarian’ judgment

Utilitarianism is the view that the right act is the one that maximizes aggregate well-being, considered from a maximally impartial perspective that gives equal weight to the interests of all persons, or even all sentient beings (Singer, 1979). This radical and demanding view is the positive core of utilitarianism. Our results suggest that so-called ‘utilitarian’ judgments in sacrificial dilemmas are *not* driven by this utilitarian aim of impartially maximizing aggregate welfare. This is not entirely surprising. It is more plausible that when individuals endorse sacrificing one person to save five others, they are following, not this demanding utilitarian ideal, but rather the more modest, unremarkable, and ordinary thought that it is, *ceteris paribus*, morally better to save a greater number (Kahane, 2012, Kahane, 2014). That everyday view involves no demanding commitment to always maximize aggregate well-being (e.g. by being willing to sacrifice 1 to save 2, or 50 to save 51) nor—more importantly for our purposes—that we must do so in a maximally impartial manner, taking into equal account even the interests of distant strangers.

Utilitarianism also has a negative or critical component. Put simply, this component is just the claim that impartially maximizing aggregate well-being is the *whole* of morality. What follows from this is that utilitarians must reject any ‘deontological’ moral constraints on the pursuit of their positive aim. They must, for example, reject moral norms forbidding us from directly harming others in certain ways or from lying or breaking promises, even if such acts would lead to a better outcome. Utilitarians must also reject inalienable rights and considerations of distributive justice, as well as principles of desert and retribution, or of purity and hierarchy. And so on.

A utilitarian must reject all deontological constraints on the pursuit of the greater good. But, again, it is obviously a mistake to assume that if someone rejects *some* deontological norms, let alone a *single* deontological constraint relating to personal harm in a specific, unusual context, then they must also reject *all* such norms, or even many of them. For example, someone can reject a specific deontological constraint on directly harming others while still holding extreme deontological views about other moral questions (such as that lying is absolutely forbidden), or radical libertarian views about property rights. Consider an analogy: an atheist would typically rejects all religious rules, but of course the fact that someone rejects a religious rule against, say, eating pork hardly amounts to any interesting step in the direction of atheism, let alone count as an ‘atheist judgment.’ Needless to say, someone making such a judgment may in fact be a Christian fundamentalist…

Recent research on sacrificial dilemmas has overlooked these points. It has mistakenly treated the rejection (or discounting) of a single intuitive deontological constraint relating to harm in a specific, unusual context, as a significant step in a utilitarian direction, and it has mistakenly assumed that when subjects instead endorse an act that will save a larger number of lives in this special context, then this endorsement must express an impartial utilitarian concern for the greater good. Yet such supposedly ‘utilitarian’ judgments reflect only a very narrow aspect of the negative side of utilitarianism. At the same time, they may reflect little or nothing of utilitarianism’s core positive side: the moral aim of *impartially* maximizing aggregate well-being. One robust result of the present study is that there appears to be no interesting relationship between so-called ‘utilitarian’ judgment and this positive core of a utilitarian approach to ethics.

The consistent association between ‘utilitarian’ judgment and antisocial tendencies is a striking illustration of the above points. In particular, recent research has overlooked the fact that the negative dimension of utilitarianism is also shared by views that are otherwise radically opposed to it. For example, egoists also approach practical questions in a calculating, no-nonsense manner, and are quick to dismiss many common moral intuitions and sentiments. Needless to say, however, egoists utterly *reject* the positive core of a utilitarian outlook, holding instead that we should care about (and maximize) only what is in our own self-interest.^7^ One ironic implication of the results of our study is that some recent research on ‘utilitarian’ decision-making may actually have been studying the psychology of egoism!

The convergence between egoist views, associated with antisocial traits such as psychopathy, and supposedly ‘utilitarian’ conclusions will seem puzzling only if the theoretical distinctions we draw above are overlooked. It is in fact not surprising that when individuals with antisocial tendencies and egoist leanings are presented with sacrificial dilemmas in which they are forced to choose between two moral options—one based on a deontological intuition against causing harm that they don’t share, and one involving harming someone to save more lives—they would choose the latter. There is nothing to attract them to the first option, while the second at least follows the same logic they employ in their own self-centered decision-making. Yet, as we found in Study 2, the moral judgments of such individuals—judgments that the current literature classifies as ‘utilitarian’—are in fact often highly responsive to whether the sacrifice in question is in one’s own self-interest.

The positive and negative aspects of utilitarianism are of course perfectly compatible at the philosophical level. However, one intriguing possibility emerging from the present study is that these positive and negative aspects may nevertheless push in opposite directions in the psychology of the lay population. The kind of no-nonsense, tough-headed and unsentimental approach to morality that makes it easier for some people to dismiss entrenched moral intuitions may also drive them away from a more impartial, all encompassing and personally demanding view of morality, and might even lead some to skepticism about morality itself. Conversely, those who *are* more attracted to such an impartial, proto-utilitarian ethics—perhaps in part due to greater empathic concern—may also be less inclined to so easily dismiss deontological constraints on harming others.

We should again emphasize that our criticism is not that such ‘utilitarian’ judgments are not based in explicit endorsement of a utilitarian ethical theory. It is doubtful that more than a tiny minority of the lay population would explicitly endorse such a theory. Nor are we expecting ordinary individuals to judge and behave, in a wide range of contexts, in complete and consistent conformity to utilitarian theory.

Rather, what our study suggests is that—even when the antisocial dimension in ‘utilitarian’ judgment is set aside—there is no relationship between such judgment and *any kind* of increased concern for the greater good, as manifested even in very modest forms of greater altruism and impartiality, such as that involved in donating to charity part of a very small bonus. As we pointed out, such a modest donation is *very* far from the extremely demanding altruism arguably required by a fully consistent utilitarian view – potentially even requiring us to give away most of our income – yet ‘utilitarian’ judgment as commonly measured in the current literature was not associated even with such a modest altruist tendency.

### 6.2 Leaving ‘utilitarian’ judgment behind

There is by now a large literature that refers to judgments endorsing sacrificial acts in classical moral dilemmas as ‘utilitarian.’ We recognize that this terminology is strongly entrenched. But the results of the present study, and the conceptual considerations we have spelled out above and in other work (Kahane, 2012, Kahane, 2014, Kahane and Shackel, 2010, Kahane et al., 2012), strongly suggest that this terminology is highly misleading.

First, it describes a tendency that is specific to an extremely unusual moral context in a way that suggests a generality that is not really there: what the current literature describes as a ‘utilitarian’ bias is in fact associated with greater *rejection* of paradigmatic utilitarian views and attitudes in other moral contexts.

Second, it implies that ‘utilitarian judgment’ and ‘utilitarian decision-making’ refer to a unitary psychological phenomenon, which may even be based in a specific neural subsystem (Greene et al., 2004) and which can be investigated by studying sacrificial dilemmas. Our results cast doubt on this assumption and suggest that, in the psychology of non-philosophers, different aspects of a utilitarian moral outlook often come apart, and may even be in some tension.

Finally, this terminology may be misleading *even* in the narrow context of sacrificial dilemmas. While choosing to push someone off a footbridge to save five is in line with a utilitarian outlook, it does not automatically follow that such a choice is driven by genuine utilitarian considerations. In fact, in the present study we found that such judgments are often driven by an outlook that is diametrically opposed to a truly utilitarian ethics.

Earlier research has suggested that ‘utilitarian’ judgment in standard moral dilemmas is uniquely associated with effortful deliberation and explicit reasoning (Greene et al., 2004). This association that has been taken to show that such judgments are more ‘rational,’ and therefore speak in favor a utilitarian approach to ethics (Greene, 2008, Singer, 2005). A growing body of research, however, has begun to tie these very same ‘utilitarian’ judgments to antisocial traits such as psychopathy and reduced empathic concern (Bartels and Pizarro, 2011, Glenn et al., 2010, Koenigs et al., 2012, Wiech et al., 2013), which are far less flattering connections. But true utilitarians should neither cheer the supposed tie between ‘utilitarian’ judgments and ‘rational’ deliberation, nor feel discomfort about the more sinister association with psychopathy—for, contrary to appearances, so-called ‘utilitarian’ response to sacrificial moral dilemmas appear to have little to do with genuine utilitarianism.

## Footnotes

2 There is more than one form of utilitarianism. However, the literature on utilitarian judgment in current moral psychology assumes a simple form of what philosophers call Act Utilitarianism (see e.g. Cushman et al., 2010, Greene, 2008)—a view broadly similar to that associated with utilitarians like Peter Singer. When we refer to utilitarianism, we shall therefore mean only something like this view. In fact on some other forms of utilitarianism (e.g. rule utilitarianism) it would not be obvious that one should, e.g., push an innocent person to his death in order to save a greater number (see Kahane & Shackel, 2010).

3 Since it is unlikely that ordinary folk follow any explicit theory, we cannot find out whether they are concerned about the greater good simply by asking them. Conversely, people may find abstract utilitarian principles attractive because they fail to see that such principles have counterintuitive and disturbing implications in many specific cases. See Lombrozo (2009) for evidence of a gap between such abstract endorsement and concrete judgment.

4 For this study and all others reported in this paper, we conducted analyses exploring whether our exclusion criteria unduly affected results. Comparisons of results using all participants and our final sample revealed no difference in the pattern of results upon excluding participants who did not complete the survey properly. Therefore, all analyses reported in this study are conducted with the final clean sample.

5 In the interests of conciseness and clarity we only report in the text those correlations that are essential to the hypotheses tested, displaying all other correlations in Table 1. All means and SDs for items across the four studies can be seen in the Supplementary materials.

6 Gleichgerrcht and Young (2013) reach the opposite conclusion after finding no correlation between ‘utilitarian’ judgment and views about an immoral selfish act. However their design employed only a single immoral vignette and a single personal dilemma which, moreover, involved an especially extreme scenario.

7 Interestingly, in Study 4 we found a further point of convergence: both utilitarians and individuals with antisocial traits tend to discount the importance of personal ties to family and country. The difference is that egoists discount these ties in favor of self-interest, whereas utilitarian discount them in favor of the greater good.
