# Supplementary material for: ‘Utilitarian’ judgments in sacrificial moral dilemmas do not reflect impartial concern for the greater good
Source: Cognition. 2015 Jan;134:193–209. doi: 10.1016/j.cognition.2014.10.005 (PMC4259516; doi:10.1016/j.cognition.2014.10.005)
Supplement: Supplementary data 1 [file mmc1.pdf]

Supplementary Materials for

'Utilitarian' Judgments in Sacrificial Moral Dilemmas Do Not Reflect Impartial Concern for the Greater Good

Guy Kahane[a,b*], Jim A.C. Everett[a c*], Brian D. Earp[a], Miguel Farias[d], Julian Savulescu[a,b]

[a] Oxford Uehiro Centre for Practical Ethics, University of Oxford, Littlegate House, St. Ebbe's St., Oxford, OX1 1PT, UK

[b] Oxford Centre for Neuroethics, University of Oxford, Littlegate House, St Ebbe's Street, Oxford OX1 1PT, UK

[c] Department of Experimental Psychology, University of Oxford, South Parks Road, Oxford, OX1 3UD, UK

[d] Centre for Research in Psychology, Behaviour & Achievement, Coventry University, Priory Street, Coventry CV1 5FB.

[*] These authors contributed equally.

Study 1 Sacrificial Personal Dilemmas ***(from Moore et al. 2008)***

Footbridge. A runaway trolley is heading down the tracks toward five workmen who will be killed if the trolley proceeds on its present course. You are standing next to the track on which the trolley is traveling, but you are too far from the workmen to warn them of the impending danger. Next to you there is a very large stranger who is minding his own business. It occurs to you that if you pushed this person onto the tracks in front of the trolley, it would stop the trolley and save the five workmen from certain death. However this will most certainly kill the stranger. *Should you push the stranger to save the workers?*

Epidemic. A viral epidemic has spread across the globe killing thousands of people. You are a medical researcher and have developed two substances in your laboratory. You know that one of them is the vaccine, but you don't know which because both of the vials have been mislabeled as a vaccine. You also know that the other substance is deadly. Once you figure out which substance is the vaccine you can create more to save thousands of lives. You have two lab assistants who work with you, and the only way to identify the vaccine with certainty is to inject the two substances into these people against their wishes. One person will live, one person will die, and you will be able to start saving lives with your vaccine. *Should you inject the lab assistants with the substances to discern which one is the vaccine?*

Soldiers. You are the leader of a small group of soldiers, and all of you are out of ammunition. You are on your way back from a completed mission deep in enemy territory when one of your men steps in a trap set by the enemy. His leg is badly injured and caught in the trap. You cannot free him from this trap without killing him. However, if you leave him behind, the enemy troops will find him and torture him to death. The soldier begs you not to leave him behind to be cruelly tortured to death. The enemy troops are closing in on your position. It is not safe for you or your men to remain with your trapped comrade any longer. In order to prevent this mans needless suffering at the hands of the enemy, you could kill him yourself by stabbing him in the heart. *Should you stab the man in the heart to prevent his suffering at the hands of the enemy?*

Hospital. You are an orderly in a hospital during the night shift. You notice that a chemical has just been spilled in a room containing six patients. This chemical is highly toxic and if left on the floor will rapidly evaporate creating a poisonous gas. This will inevitably kill all of the patients in the room. This chemical cannot simply be mopped up, nor can it be soaked up with towels or sheets. However, because you have worked around this chemical before, you know that this chemical absorbs into human skin very rapidly. You could pull one

of the patients out of bed onto the spill so that the chemical will completely soak into this persons skin instead of evaporating into the air in the room. This will quickly kill the one patient but save the other five patients from the gas. *Should you kill this patient to save the others?*

Study 2 Sacrificial Dilemmas

Self-Beneficial Personal Dilemmas (from Moore et al. 2008)

Burning Building. You and five other people are trapped in a burning building. There is only one emergency exit through which all of you could escape to safety, but it is blocked by burning debris. You notice another person in the hallway leading to the exit who has been injured but is about to crawl to safety through a small hole at the bottom of the exit door. You and the five people behind you do not have time to climb through the small hole. You realize that you could grab the injured man and use his body as a battering-ram to break through the burning blockage in the hallway that is preventing your escape. Doing this is certain to kill him. However, if you do not do this, you and the five people behind you will die. *Should you kill this man in order to save yourself and the other five people?*

Modified Crying Baby. Enemy soldiers have taken over your village. They have orders to kill all remaining civilians over the age of two. You and some of your townspeople have sought refuge in two rooms of the cellar of a large house. Outside you hear the voices of soldiers who have come to search the house for valuables. Your baby, who is with you in the room, begins to cry loudly. You put your hand over his mouth to block the sound. If you remove your hand from his mouth his crying will summon the attention of the soldiers who will spare your baby's life, but will kill you and the others hiding in both rooms. To save yourself and the others you must keep your hand on his mouth and smother your baby to death. *Should you smother your baby in order to save yourself and the other townspeople?*

Modified Submarine. You are a crewperson on a marine-research submarine traveling underneath a large iceberg. An onboard explosion has damaged the submarine, killed and injured several crewmembers. Additionally, it has collapsed the only access corridor between the upper and lower parts of the submarine. The upper section, where you and most of the others are located, does not have enough oxygen remaining for all of you to survive until you reach the surface. Only one remaining crewmember is located in the lower section, where there is enough oxygen. There is an emergency access hatch between the upper and lower sections of the submarine. If released, it will fall to the deck and allow oxygen to reach the upper section. However, a crewmember in the lower section was knocked

unconscious and is lying beneath the hatch while you and the rest of the crew are almost out of air. If you shove the hatch open you and the others will have air, but it will fall to the deck, crushing the unconscious crewmember. *Should you open the hatch and crush the crewmember below to save yourself and the other crewmembers?*

Preventing Ebola. You are a Peace Corps health-worker who is volunteering in a rural African village. A prominent man from a nearby village has contracted an Ebola virus that is extremely contagious, incurable, and almost always fatal within a week. Miraculously, this man has survived for a month, and so he must be a rare carrier who is immune to the virus' deadly effects. However, this man wrongly believes that your health center can cure his disease. You see him approaching and you know that if he enters the village he will spread the virus to hundreds of innocent people who, unlike him, will die. There is a loaded gun in the health center. You realize that the only way you can prevent him from entering the village and spreading the virus to you and the rest of the village is to shoot and kill him as he approaches. *Should you kill the man in order to save yourself and the rest of the village?*

Other-Beneficial Dilemmas (from Moore et al. 2008)

On the Waterfront. You are part of a shipyard dock team that attaches crane cables to huge transport containers that are to be unloaded from ships onto the shore. You and the others attach these cables and then ride on top of the containers, wearing safety harnesses, to make sure that the containers are unloaded properly. While you are riding on top of one container that is just being lifted out of the cargo bay, you see the red warning light that indicates that the crane cable is about to fail. You realize that if the cable fails and the container falls onto the deck of the ship, many of the crewmembers below will be crushed to death. You see that the cable is in danger because two other crewmembers are fighting on top of the container, causing it to sway dangerously. You could run over to the fight and push them apart, but one of the crewmembers has taken off his safety harness, and will certainly fall to his death. However, if you do not do this, the continued swaying will cause the cable to fail and the container to fall onto the deck, killing several people below. *Should you push the un-harnessed crewmember off the container to his death to save the people below?*

Modified Vaccine Test. A viral epidemic has spread across the globe killing thousands of people. You are a medical researcher and have developed two substances in your laboratory. You know that one of them is a vaccine, but you don't know which because both of the vials have been mislabelled as vaccine. You also know that the other substance is deadly. Once you figure out which substance is the vaccine you can create more to save thousands of lives. You have two lab assistants who work with you, and the only way to

identify the vaccine with certainty is to inject the two substances into these people against their wishes. One person will live, the other will die, and you will be able to start saving lives with your vaccine. *Should you kill one of these people with a deadly injection in order to identify a vaccine that will save thousands of lives?*

Modified Footbridge. A runaway trolley is heading down the tracks toward five workmen who will be killed if the trolley proceeds on its present course. You are standing next to the track on which the trolley is traveling, but you are too far away from the workmen to warn them of the impending danger. Next to you there is a very large stranger who is minding his own business. It occurs to you that if you pushed this person onto the tracks in front of the trolley, it would stop the trolley and save the five workmen from certain death. However, this would most certainly kill the stranger. *Should you push this stranger onto the tracks to save the five workmen?*

Bike Week. You are an expert motorcycle rider and you have gone on vacation in order to participate in Bike Week. Thousands of other motorcycle riders from across the country have come to ride in this event. As you are riding down the road in the front of a large group of other riders you see that someone up ahead is losing control of their bike. As you speed up to pull alongside the unstable rider, you realize that this person is going to crash at any second. This would certainly result in a large pile-up and several deaths as the riders behind you run over each other trying to avoid the crashed rider. You realize that you could physically run this rider off the road and into some trees. This would cause him to crash and, at your current speed, almost certainly die, but it would prevent a crash in the middle of the street and the large pile-up of riders behind you. *Should you crash the other rider to avoid the deaths of the riders behind you?*

Study 2 Supplementary Method

Hypothetical Donation Vignette

For this item, participants were asked to:

> "Imagine that you have just received a bonus at work of $100. Your company, however, has a policy for all staff members that they can either take this bonus money of $100 for themselves with no penalties, or choose to donate it to a respected charity that helps people in the Third World. You can choose to keep or donate as much of the bonus as you wish. If you choose to donate the bonus, the company will double what you put in: if you donate £50 then the company will donate £100, and if you donate $100 the company will donate $200. This choice is made using an

anonymized online system, so no one in the company will know what you decided to do."

Participants were then asked how much of the bonus they would donate, indicating their answer on a sliding scale from $0-100.

Self-Sacrifice Dilemma

In Study 2, we also introduced a variant of the classic Switch dilemma (Foot, 1969). In its standard version, participants are asked whether one should switch a runaway trolley that is on course to kill five innocent workers, thereby shifting it to another track on which it will instead kill only a single person. We included a modified version where participants had a third option—to divert the trolley onto a third track, leading to *their own* death (Thomson, 2008; see Huebner & Hauser, 2011). In this Self-Sacrifice variant, participants who choose to save the five must choose whether to sacrifice themselves or another person.[1] Huebner & Hauser (2011) report that a majority of subjects endorsed self-sacrifice in this dilemma. We made the following predictions: (a) that such self-sacrifice would *not* be correlated with rates of 'utilitarian' judgment in personal dilemmas; (b) that it will be *negatively* correlated with psychopathy and egoism; and (c) that, by contrast, it will be *positively* correlated with Identification With All of Humanity.

Study 2 Supplementary Results

Self-Sacrifice Dilemma

Descriptive statistics revealed that in the self-sacrifice version of the Switch dilemma, most participants chose the self-sacrifice option (49%), with the next most common option being to sacrifice a stranger to save the life of the five (30%), with the remaining participants (21%) choosing to do nothing, meaning that the five people would die. ANOVAs were then conducted to investigate the relationship between individual differences and standard 'utilitarian' judgements with responses on the self-sacrifice version.[2]

Next, we investigated how people who endorse the 'utilitarian' action in standard sacrificial dilemmas respond when self-sacrifice is an option. To test this we used a one-way

---

[1] Note that although from a utilitarian standpoint one should choose to save the five in this dilemma, it is arguably less clear whether, from an impartial utilitarian standpoint, one should sacrifice oneself rather than the other person. However, although offering a choice to either let the five die or sacrifice oneself would have been a sharper test of willingness to endorse a utilitarian judgment that goes against self-interest, responses to such a measure would have been biased by the desire of subjects to appear consistent with their responses on the standard personal dilemmas.

between subjects ANOVA with bonferroni correction to explore whether a 'utilitarian' tendency in sacrificial personal dilemmas was associated with a distinctive pattern of response in the self-sacrifice dilemma (No action = 60; other sacrifice = 87; self sacrifice = 139). Results of the ANOVA revealed that different responses in the new self-sacrifice dilemma were associated with different rates 'utilitarian' judgements in the standard dilemmas (F (2, 283) = 16.84, $p < .001$), with the highest rate of 'utilitarian' judgements for those who endorsed the other-sacrifice option in the self-sacrifice dilemma, followed by those who endorsed self-sacrifice, and the lowest 'utilitarian' judgements in the standard dilemmas amongst those who endorsed doing nothing in this new dilemma. That is, people who are more 'utilitarian' in the standard dilemma also tend to favour sacrificing another person over oneself. Further, there was a significant difference in standard 'utilitarian' answers between those who chose to sacrifice the other individual on one hand, and both other choices ($p < .001$): people who endorse the 'utilitarian' action in the standard dilemmas favour sacrificing another person even when self-sacrifice is an option. There were, however, no significant differences in rates of 'utilitarian' judgement in standard dilemmas between those who opted to do nothing or sacrifice themselves: people who chose to do nothing or sacrifice themselves in the self-sacrifice dilemma were not significantly more likely to endorse the 'utilitarian' action in the standard dilemmas.

A second one-way between-subjects ANOVA (psychopathy: high, low) using a median split with bonferonni correction revealed a significant difference in responses between individuals high and low on psychopathy in the self-sacrifice dilemma (F (1, 284) = 9.98 p = .002), with individuals high in psychopathy being significantly more likely to sacrifice the other individual (42%), and individuals low in psychopathy being significantly more likely to sacrifice themselves (63%). There was no difference between likelihood of doing nothing between individuals high (22%) and low (20%) in psychopathy: the observed difference related to whether participants chose to sacrifice themselves or another.

Finally, another one-way between-subjects ANOVA (Identification With All of Humanity (IWAH): high, low) using a median split with bonferonni correction revealed a significant difference in responses between individuals high and low in IWAH in the self-sacrifice dilemma (F (1, 284) =4.79, p = .03), with individuals low in IWAH being just as likely to sacrifice the other individual (38%) as themselves (40%), while individuals high in IWAH were significantly more likely to sacrifice themselves (57%) than the other person (23%). As with psychopathy, there was no difference observed in the tendency to do nothing between those high (20%) and low (22%) in IWAH.

As predicted, therefore, high psychopathy participants tended to endorse the other-sacrifice option in the modified Switch dilemma, whereas low psychopathy participants were overwhelmingly more likely to endorse the self-sacrifice option. Also in line with our

predictions, IWAH was positively associated with choosing the self-sacrifice option and *negatively* associated with choosing the other-sacrifice option.

Study 3 Supplementary Method

‘Real-World Utilitarianism’ Items

Children in Poor Countries. For this item, participants were first asked to read Peter Singer’s well-known ‘pond’ example, describing a scenario in which one can easily save a drowning child, adapted from Singer (1972), before being asked to rate the wrongness of failing to help the drowning child in the pond (*1 = Not at all wrong, 5 = Very wrong*).

Participants were then asked to read a short passage about conditions for children in poor countries before being asked to rate the wrongness of failing to donate money to help children in poor countries (*1 = Not at all wrong, 5 = Very wrong*):

> “Many thousands of children in poor countries die everyday because they don’t have enough to eat, because of poor sanitation, or because their parents can’t afford medical treatment. Assume that aid organizations are working effectively to reduce poverty and provide clean water and basic health, and that these efforts are saving lives. If these organizations had more money, they could do more, and more lives would be saved. By donating a relatively small amount of money you could save the life of a child in a poor country.”

Obligations of the Wealthy in the West. Participants were asked whether they “think that well-off people in the West have moral obligations to help poor people in developing countries” (*1 = It would be wrong for well-off people in the West to help poor people in developing countries; 3 = Well-off people in the West don’t have to help poor people in developing countries, but it would be nice if they did; 5 = Well-off people in the West must help poor people in developing countries;* i.e. higher scores indicate greater obligations to help, lower scores indicate weaker (or no) obligations to help).

Help Poor Foreign Country over their Own. Participants were asked “If you could help people in need from your own country, or people in great need in a very poor foreign country, which would you choose?” (*1 = Always people from my own country; 3 = Both equally; 5 = Always people from a very poor foreign country*; i.e. lower scores indicate thinking it is better to help people from your own country).

Concern for Future Generations. Participants were asked to rate the extent to which they thought that people now have an obligation to make sacrifices in the present to prevent climate change that would cause great harm to future generations (*1 = Definitely No, 5 = Definitely Yes*; i.e. higher scores indicate stronger commitment to climate change action).

Study 3 'Real-World' Harm Items

Animal Experimentation. Participants were asked to rate how acceptable or wrong they thought it was to use animals in experiments that cause the animals pain or serious harm (*1 = Perfectly Acceptable; 4 = Somewhat wrong; 7 = Deeply wrong*).

Abortion. Participants were asked to rate how acceptable abortion is on a 1-7 scale (*1 = Perfectly acceptable; 4 = Somewhat wrong; 7 = Deeply wrong*).

Wrongness of Eating Meat. Participants were asked to rate how morally wrong or right eating meat is on a 1-7 scale (*1 = Perfectly Fine; 4 = Neither fine nor wrong, 7 = Deeply Wrong*).

Torture. Participants were asked to rate what they thought about torture (*1 = It is always wrong to use torture; 2 = Torture is a bad thing, but in special cases where torture might save the lives of many innocent people, we sometimes have to allow it; 3 = Torture is often a legitimate method of interrogation*).

Study 4 'Greater Good' Dilemmas

Benjamin's Car or Donation. Benjamin is a college student who has always wanted to have his own car. He works after school jobs, saves all of his money, and rarely goes out, until he has $7000. On his way to the used car dealership, he reads about a Tsunami in South East Asia that has left thousands dead, wounded and homeless. A number is given where donations can be processed to help these victims. Benjamin knows that if he were to donate a significant amount to this charity, his donation would make a real difference to the lives of several of the victims of the Tsunami. *How wrong would it be for Benjamin to go on and buy the car, rather than donate any of the money?*

Anti-Malaria Foundation. John gets a letter from the "Against Malaria Foundation" in the mail, telling him that a donation of $100 will save a child's life. Although he has no doubt that this donation will indeed save a child's life and make the world a better place, John also believes that he has a right to give priority to his own interests and happiness. John wants to spend the money on a new mobile phone, and is therefore considering throwing the envelope away. *How wrong would it be for John to throw the envelope away and buy a new mobile phone?*

Janet and her Mother. Janet's mother is feeling really lonely and would like Janet to come and spend the day with her. Janet is an engineer who had planned on using the day to volunteer for Habitat for Humanity, a volunteer organization that builds houses for poor families. By volunteering, she would be helping a whole family in need for a long period, rather than cheering up her mother for one day. *How wrong would it be for Janet to visit her mother instead of going on to volunteer?*

Vegetarianism. Kathleen has recently read a book that describes the methods of modern factory farming in gruesome detail. She has been persuaded by the argument of the book that by eating meat, we are causing many animals to undergo great suffering. This suffering could be reduced if many of us became vegetarian. Although Kathleen agrees that we could significantly reduce animal suffering by becoming vegetarian, she also thinks that humans are more important than animals, and that it is natural for us to eat meat. *How wrong would it be for Kathleen to continue to eat meat?*

One vs. Many Donation. Mark is a US businessman who would like to give $1,000 to charity, and is deciding between two charities. The first focuses on preventing disease in the US, and Mark's donation would save the life of one child. The second focuses on

preventing disease in a distant foreign country Mark has never been to, and his donation would save the lives of several children. Mark has already made up his mind to donate to one of the two charities, and just needs to decide to which one. *How wrong would it be for Mark to donate to the charity in his own country?*

Veronica's Comfortable Lifestyle. Veronica is a writer who has written several successful popular books, and therefore has a rather ample income. She has worked hard for this income, and feels proud of her accomplishments. Veronica has enough money to live a comfortable life, with money left over for things like vacations and even the latest gizmos and gadgets – the fruit of many years of scholarly labor. Veronica realizes that she could survive at a decent level of happiness with much less money, and she knows that if she were to give away large sums of money to effective charities, she could actually save dozens of people from squalor and even death. But Veronica would have to give up the cozy life she's worked so hard for and live very modestly. *How wrong would it be for Veronica to continue to enjoy her comfortable life instead of giving away large sums of money to charity?*

Firefighter. Albert is a firefighter who is trying to rescue people from a burning building. The building is about to collapse and in the time left, Albert will only be able to rescue one more person. In the room he has entered, Albert finds two trapped people that he immediately recognizes. One is a famous peace negotiator well known for his work resolving armed conflicts around the world and who is likely to continue doing this important work if he survives. The other is a poor, uneducated housekeeper. The housekeeper is Albert's mother. Albert has to decide which of these to save, and the one he does not save will die. *How wrong would it be for Albert to save his mother?*

Supplementary References

Huebner, B. & Hauser, M. (2011). Moral judgments about altruistic self-sacrifice: When philosophical and folk intuitions clash, *Philosophical Psychology*, *24(1)*, 73-94.

Thomson, J. J. (2008). Turning the Trolley. *Philosophy and Public Affairs. 36(4),* 359-374.
